# Rapid Processing of Observed Touch through Social Perceptual Brain Regions: An EEG-fMRI Fusion Study

**DOI:** 10.1523/JNEUROSCI.0995-23.2023

**Published:** 2023-11-08

**Authors:** Haemy Lee Masson, Leyla Isik

**Affiliations:** ^1^Department of Psychology, Durham University, Durham DH1 3LE, United Kingdom; ^2^Department of Cognitive Science, Johns Hopkins University, Baltimore, Maryland 21218

**Keywords:** EEG-fMRI fusion, social perception, social touch observation, somatosensory simulation

## Abstract

Seeing social touch triggers a strong social-affective response that involves multiple brain networks, including visual, social perceptual, and somatosensory systems. Previous studies have identified the specific functional role of each system, but little is known about the speed and directionality of the information flow. Is this information extracted via the social perceptual system or from simulation from somatosensory cortex? To address this, we examined the spatiotemporal neural processing of observed touch. Twenty-one human participants (seven males) watched 500-ms video clips showing social and nonsocial touch during electroencephalogram (EEG) recording. Visual and social-affective features were rapidly extracted in the brain, beginning at 90 and 150 ms after video onset, respectively. Combining the EEG data with functional magnetic resonance imaging (fMRI) data from our prior study with the same stimuli reveals that neural information first arises in early visual cortex (EVC), then in the temporoparietal junction and posterior superior temporal sulcus (TPJ/pSTS), and finally in the somatosensory cortex. EVC and TPJ/pSTS uniquely explain EEG neural patterns, while somatosensory cortex does not contribute to EEG patterns alone, suggesting that social-affective information may flow from TPJ/pSTS to somatosensory cortex. Together, these findings show that social touch is processed quickly, within the timeframe of feedforward visual processes, and that the social-affective meaning of touch is first extracted by a social perceptual pathway. Such rapid processing of social touch may be vital to its effective use during social interaction.

**SIGNIFICANCE STATEMENT** Seeing physical contact between people evokes a strong social-emotional response. Previous research has identified the brain systems responsible for this response, but little is known about how quickly and in what direction the information flows. We demonstrated that the brain processes the social-emotional meaning of observed touch quickly, starting as early as 150 ms after the stimulus onset. By combining electroencephalogram (EEG) data with functional magnetic resonance imaging (fMRI) data, we show for the first time that the social-affective meaning of touch is first extracted by a social perceptual pathway and followed by the later involvement of somatosensory simulation. This rapid processing of touch through the social perceptual route may play a pivotal role in effective usage of touch in social communication and interaction.

## Introduction

Touch evokes a strong social and emotional response in third-party observers as well as the direct recipient (Hertenstein et al., 2006a, 2009). During mere observation, humans accurately extract the social-affective meaning of a touch gesture, with high interobserver reliability (Lee Masson and Op de Beeck, 2018). For example, it is easy to understand how a warm embrace between a couple can be pleasant and emotionally arousing, while an accidental push from a stranger in a line can be unpleasant but not as arousing. The functional magnetic resonance imaging (fMRI) literature suggests viewing social touch increases posterior insula responses to observed social touch (Morrison et al., 2011; but see Ebisch et al., 2011) and leads to shared somatosensory responses between self-experienced and observed social touch (Ebisch et al., 2008, 2011; Gazzola et al., 2012). Further, the social-affective meaning of observed touch is represented in social-cognitive brain areas, including temporoparietal junction and posterior superior temporal sulcus (TPJ/pSTS), as well as somatosensory cortex, and observing social touch leads to enhanced functional communication between these regions (Lee Masson et al., 2018, 2020).

These results suggest that observed touch is understood not only from direct perceptual signals, but also via somatosensory simulation (for review, see Peled-Avron and Woolley, 2022). Somatosensory simulation has been shown to vary greatly between individuals based on the degree of emotional empathy, attitude toward social touch, and autistic traits (Gallese and Ebisch, 2013; Giummarra et al., 2015; Peled-Avron et al., 2016; Peled-Avron and Shamay-Tsoory, 2017; Lee Masson et al., 2018, 2019). However, recent work has called into question the direct role of simulation in other aspects of social perception like action recognition (Caramazza et al., 2014). Because of the slow temporal resolution of fMRI, it is difficult to understand the direction of information flow between somatosensory and social perceptual brain regions.

Prior electroencephalogram (EEG) studies have investigated the neural processing of social touch observation, mostly focusing on the μ rhythm indexing somatosensory simulation and event-related potentials (ERPs; Peled-Avron et al., 2016; Schirmer and McGlone, 2019; Addabbo et al., 2020). A few prior studies have provided initial insight into how fast the brain processes an observed touch event, and found the observation of another person receiving simple, nonsocial touch, such as a paintbrush touching a hand, evoked early involvement of somatosensory ERPs (Adler and Gillmeister, 2019; Rigato et al., 2019a,b). In contrast, adding social-affective complexity to a touch scene results in longer processing time reflected by increases in P100 and late positive potential (Peled-Avron and Shamay-Tsoory, 2017; Schirmer and McGlone, 2019). However, no prior study on social touch has directly linked stimulus features to EEG timeseries, so it remains to be seen how quickly visual and social-affective features of observed touch are processed. Furthermore, although the involvement of the somatosensory cortex has been suggested in these EEG studies, spatial localization with EEG is often inconclusive.

Here, we ask whether social touch features are processed via social perception or somatosensory simulation. To answer this question, we apply new methods in fMRI-EEG fusion (Cichy and Oliva, 2020), and use representational similarity analysis (RSA) to link stimulus features, fMRI multivoxel patterns, and EEG activity patterns from observed touch scenes. In particular, we examine how fast each social-affective feature is processed, as well as the time course of feature representations in different brain regions, including early visual cortex (EVC), TPJ/pSTS and somatosensory cortex. If somatosensory simulation drives social touch perception, we would expect the neural patterns from somatosensory cortex to correlate with earlier EEG activity patterns than TPJ/pSTS. However, we find the opposite results: EEG signals correlate first with EVC followed by TPJ/pSTS, and finally somatosensory cortex. We further find that while EVC and TPJ/pSTS each share unique variance with EEG neural patterns and social-affective features, somatosensory cortex does not. Together, these results indicate that social-affective features in observed touch scenes are directly extracted via a social perceptual pathway without direct somatosensory simulation.

## Materials and Methods

### Participants

A total of 21 participants (male = 7, mean age = 20.9 years, age range 18–32 years) took part in the EEG study. They all reported normal or corrected-to-normal vision. A total of 20 participants were recruited through the Johns Hopkins University SONA psychological research portal and received research credits as compensation for their time. One participant was compensated with a monetary reimbursement. They provided written informed consent before the experiment. The study was approved by Johns Hopkins University Institutional Review Board (protocol number HIRB00009835).

### Stimuli

**Figure 1.**
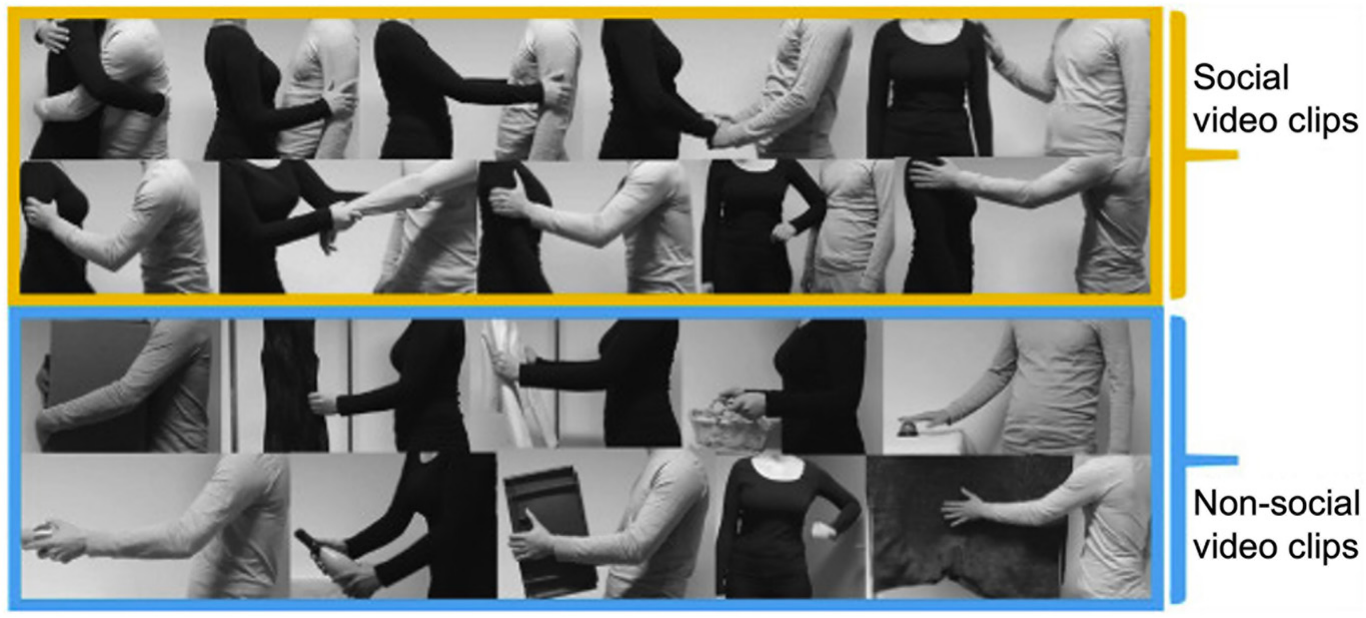
A few example frames of video clips in two categories (social and nonsocial). Images in a yellow box show representative frames of social touch videos. The top row shows examples of pleasant touch events and the second row unpleasant touch events. Frames of nonsocial touch videos displaying matched human-object interactions are shown in a blue box. This figure is published in compliance with a CC-BY-NC-ND license (https://creativecommons.org/licenses/by-nc-nd/4.0/) and is re-used from Figure 1 in the original study (Lee Masson et al., 2018). The complete set of original video materials is available at https://osf.io/8j74m/.

We used a stimulus set developed and validated in a previous study (Lee Masson and Op de Beeck, 2018). The original stimulus set consisted of 39 social and 36 nonsocial 3-s videos. Social videos showed 18 pleasant, three neutral, and 18 unpleasant human-human touch interactions, such as hugging or slapping a person. Nonsocial videos showed human-object touch manipulation that are matched to the social videos in terms of biological motion, such as carrying a box or whacking a rug (Fig. 1).

For the current study, all videos were trimmed to a duration of 0.5 s centered around the touch action to improve time-locking to the EEG signal. Each video contained 13 frames of 720 (height) × 1280 pixels in size. To ensure trimming the videos did not alter the perceived valence and arousal of touch events, we had human annotators from the online platform Amazon Mechanical Turk rate the valence and arousal of each video. We found ratings were highly correlated between the trimmed and full-length videos (Spearman *r* = 0.96 for valence and 0.94 for arousal).

### EEG experimental procedure and design

During the EEG recording, participants were seated comfortably on a chair and viewed the videos displayed on a back-projector screen in a Faraday chamber (visual angle: 15 × 13°, distance from the screen: 45 cm). They were instructed to view each video and press a button on a Logitech game controller when they detected two consecutive videos that were identical (One-Back task). These catch trials, involving participants' motor responses, were excluded from the analysis. This orthogonal task aimed to get participants to pay attention to the videos. Each block (*N* = 15) consisted of 83 trials, with the 75 videos presented once in a pseudo-random order and four catch trials (i.e., four randomly selected videos were shown twice).

The videos were shown using an Epson PowerLite Home Cinema 3000 projector. A light-sensitive photodiode was used to track the onset and offset of video presentation on the projector screen to account for delays between the time that the computer-initiated stimulus presentation and the time that the stimulus was displayed. Each trial started with a black fixation cross on a white background screen, shown for a random duration between 1 and 1.5 s, followed by a 0.5-s video. The total duration of each block was ∼2.4 min. After each block, participants were encouraged to take a break for as long as needed before continuing with the following block. The experiment consisted of 1245 trials and took <1 h, including breaks.

The experiment was run using the Psychophysics Toolbox (Brainard, 1997; Pelli, 1997; Kleiner et al., 2007) in MATLAB (R2020a, The Mathworks). The EEG experiment employed a within-subject design, with EEG neural activity as the dependent variable, and stimulus features and fMRI neural patterns as the independent variables.

### EEG acquisition and preprocessing

During the experiment described above, the EEG data were continuously collected with a sample rate of 1000 Hz using a 64-channel Brain Products ActiCHamp system with actiCAP electrode caps (Brain Products GmbH). An electrolyte gel was applied to each electrode to improve impedances. We aimed to keep electrode impedances below 25 kΩ throughout the experiment. The Cz electrode acted as an online reference.

MATLAB R2020a and the FieldTrip toolbox were used for EEG data preprocessing (Oostenveld et al., 2011). First, we corrected for lags between the stimulus triggers and the stimulus presentation on the projector screen by aligning the EEG data to the stimulus onset defined by the photodiode. The data were segmented into 1.2-s epochs (0.2 s prestimulus to 1 s poststimulus onset). Next, the data were baseline-corrected using the 0.2 s prestimulus and high pass filtered at 0.1 Hz to remove slow drifts.

For artifact rejection, we discarded bad channels and trials contaminated with muscle or eye artifacts. Data were bandpass filtered from 110 to 140 Hz, and a Hilbert transformation was applied. Timepoints with a z-value above 15 were considered to belong to muscle artifacts and removed. In addition, channels and trials with high variance were manually rejected using the ft_rejectvisual function in FieldTrip. Afterward, independent component analysis was performed to detect eye movement components and remove eye artifacts from the data. Catch trials and any trials with participants' motor responses were excluded from the analysis. This preprocessing step yielded, on average, 1122 ± 50 trials and 62.6 ± 1.27 channels. Only participants with no more than six channels removed were kept, resulting in one participant being excluded from further analysis. Lastly, preprocessed data were re-referenced to the median across all channels, low-pass filtered at 100 Hz, and downsampled to 500 Hz.

### Event-related potential analysis

We performed ERP analysis to investigate when and where the brain shows different neural responses to social versus nonsocial touch. ERPs from all trials were averaged for each condition and participant using the ft_timelockanalysis function in the FieldTrip toolbox. For group-level analysis, the grand averaged ERPs over participants for each condition were computed using the ft_timelockgrandaverage function. As described above, noisy channels were excluded from each participant's data, and group-level ERP analysis included 48 channels common to all 20 participants. Differences between the two conditions were calculated and visualized with the ft_math and ft_topoplotER functions, respectively. For statistical inference, ERPs were averaged across successive 100-ms time slices for each channel, from 0.2 s prestimulus to 1 s poststimulus onset. A *t* test was performed with the Bonferroni correction at an α level of 0.05 to determine brain response differences between the two conditions using the ft_timelockstatistics function, and we report z scored *t* values.

### Pairwise EEG decoding analysis to construct representational dissimilarity matrices

**Figure 2.**
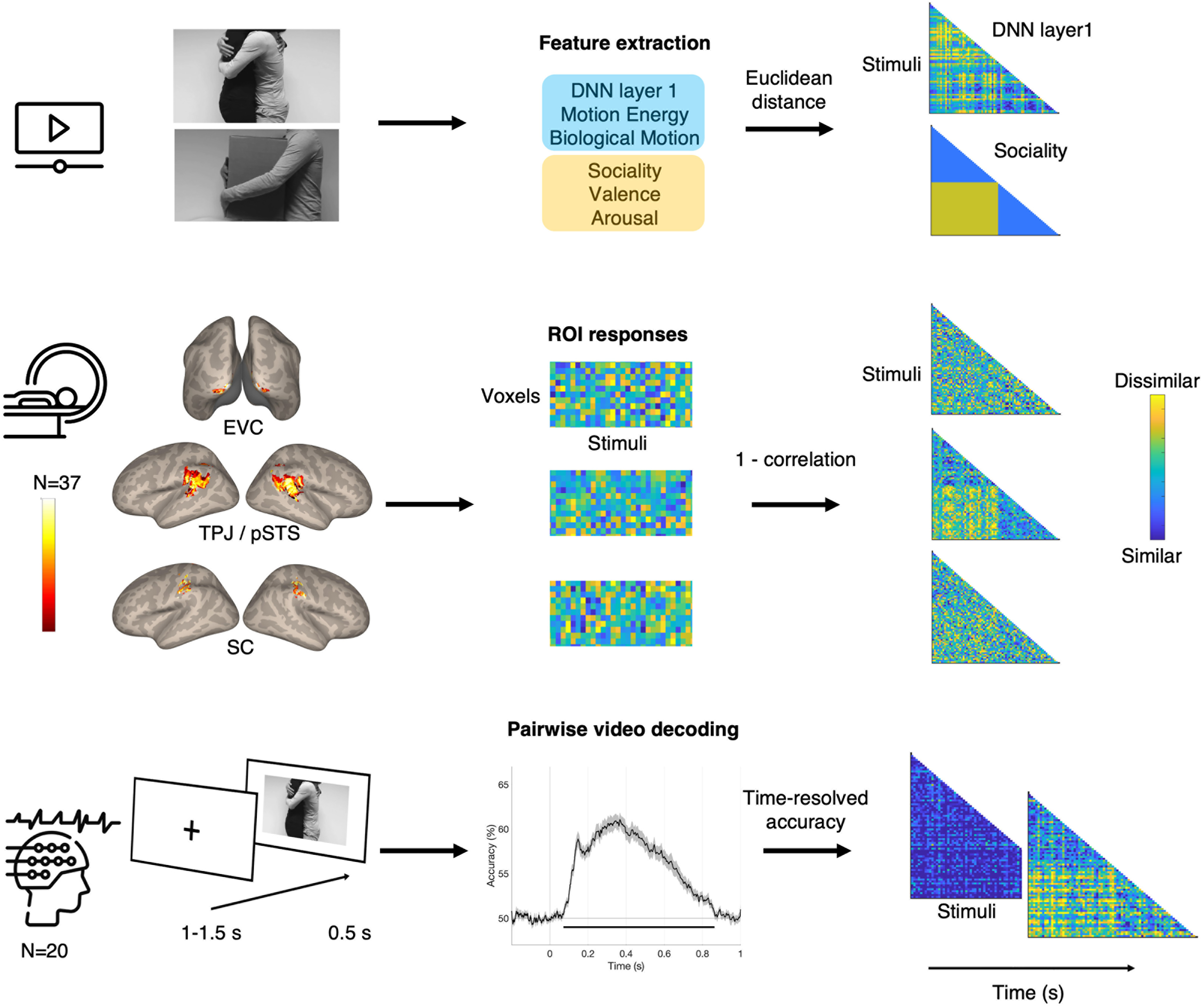
Experimental and analysis overview for evaluating how EEG signals correlate to six stimulus features as well as the fMRI multivoxel patterns of three ROIs. Top panel, Three visual and three social-affective features were extracted from each video. Euclidean distance was calculated between all pairs of stimuli to generate representational distance matrices (RDMs) for each feature. deep neural network (DNN) layer 1 and sociality RDMs are visualized. Blue in the matrices indicates high similarity, while yellow indicates high dissimilarity for each stimuli pair. Middle panel, Activated voxels during video viewing in three ROIs are shown. Voxels in bright yellow in the brain map indicate activations found in all 37 fMRI participants while voxels in dark red indicate activations found in one participant. Across these voxels, dissimilarity (1-correlation) was calculated between all pairs of stimuli to generate fMRI RDMs. RDMs of three ROIs are visualized. Bottom panel, EEG signals from 21 participants were recorded during video viewing. Pairwise decoding accuracy was calculated at each time point with a 2-ms resolution and used to generate time-resolved EEG RDMs. The horizontal line in the decoding accuracy plot marks significant time windows where observed accuracy is above chance (one-tail sign permutation for group-level statistics, cluster-corrected *p* < 0.05). EEG RDMs at 0 and 200 ms poststimulus onset are visualized. EVC = early visual cortex, TPJ/pSTS = temporoparietal junction/posterior superior temporal sulcus, SC = somatosensory cortex.

We performed time-resolved multivariate pattern analysis to link the EEG neural activity patterns to (1) stimulus features and (2) the fMRI multivoxel patterns of the early visual cortex (EVC), social brain regions: TPJ/pSTS, and the somatosensory cortex (Fig. 2). Before decoding, each participant's preprocessed EEG data were randomly split into two folds for cross-validation. To improve the signal-to-noise ratio (Dima et al., 2022), we created pseudo-trials by averaging six to eight trials corresponding to the same stimulus. Multivariate noise normalization was also performed (Guggenmos et al., 2018). Finally, time-resolved pairwise EEG decoding analysis was performed using a linear support vector machine classifier implemented in the LibSVM library (C.C. Chang and Lin, 2011). Voltages from all EEG channels were considered features at each time point. The process was repeated ten times, and decoding accuracies were averaged over all iterations for each participant. Decoding performance was used to create a time-resolved EEG neural representational distance matrix (RDM), in which high decoding accuracy results in patterns that are more different from one another while low accuracy results in patterns that are more similar (Fig. 2). Since decoding is only performed to generate the RDM, decoding results are not reported in Results. Instead, Figure 2, bottom panel, includes the group-level decoding results, averaged across all pairs of stimuli and participants. Note that the time course of group averaged decoding accuracy was similar to results observed in previous studies using visual stimuli (Isik et al., 2020; Dima et al., 2022).

### EEG to feature representational similarity analysis

Pairwise decoding results were used to generate each participant's time-resolved neural EEG RDMs, which were correlated to six stimulus features and three brain regions from the fMRI data. Features included three visual features (a low-level visual feature, motion energy, and perceived biological motion similarity between video pairs) and three social-affective features (sociality, valence, and arousal). How each of the six feature RDMs was generated is briefly described below.

(1) The low-level visual feature was extracted from the first convolutional layer of AlexNet (Krizhevsky et al., 2012), pretrained on the ImageNet dataset (Russakovsky et al., 2015), using PyTorch (version 1.4.0). Layer 1 was chosen as it captured early visual responses well in a previous EEG study that also used video clips depicting everyday actions (Dima et al., 2022). The middle frame of each 0.5-s video clip was normalized, resized to 640 × 360 pixels, and became an input to the first layer of AlexNet. The size of the kernels for the first layer is 11 × 11, resulting in an output size of 89 × 159 × 64 for each stimulus. The Euclidean distance between the resulting features for each pair of videos was used to generate the low-level visual RDM. (2) For motion energy, we estimated optical flow for each pixel of every video frame using the Farneback method implemented in MATLAB. The sum of optic flow across all pixels for each frame was calculated for each video 13 frames × 75 videos). The Euclidean distance between all video pairs was used to generate a motion energy RDM. (3) In a separate study [H Lee Masson, unpublished data; approved by the Social and Societal Ethics Committee of KU Leuven (G-2016 06 569)], 45 participants who provided written informed consent before the experiment viewed all pairs of videos and made judgments on biological motion similarity using a seven-point Likert scale (“How similar are the movements of human touches in the two videos?,” 1, very distinct; 7, identical). The pairwise similarity ratings were averaged across participants and subtracted by 7. The resulting scores were used to generate a biological motion RDM. (4) The sociality of each video, here defined as the social versus nonsocial content of touch, is a binary feature. The distance between pairs of videos from the same category was expressed as 0 (same), while videos from different categories were assigned distances of 1 (different) in the matrix. (5) For valence and (6) arousal of touch, we re-used ratings of 37 participants from our two previous studies (Lee Masson and Op de Beeck, 2018; Lee Masson et al., 2019) and generated both RDMs by calculating the absolute value of the rating differences for each pair of stimuli. Plots illustrating the sociality, arousal, and valence RDM can be found in Figure 2 of our previous study (Lee Masson et al., 2018).

A rank correlational method was used to link each participant's time-resolved EEG neural RDMs to each stimulus feature. As a follow-up analysis, we fit a multiple regression model with all six features as predictors of EEG neural patterns. Finally, a *post hoc* analysis was performed employing a multiple regression model to examine whether the features describing the body parts involved in giving and receiving touch predict neural patterns during social touch observation. Two feature RDMs were devised to capture either the body part used for initiating touch (giving touch), including the torso (with nine videos), hand (*N* = 24), arm (*N* = 3), and elbow (*N* = 3), or the body part that was touched (receiving touch), including the torso (with nine videos), arm (*N* = 24), hand (*N* = 3), and abdomen (*N* = 3). Note that these features show high correlation (*r* = 0.65).

All analyses were performed using 10-ms sliding windows with an overlap of 6 ms of EEG neural activity. We calculated leave-one-subject-out correlation where each subject's EEG signals were correlated with the group average (excluding that subject) and the average across held out subjects was used as a measure of the noise ceiling. (Nili et al., 2014).

### EEG to fMRI representational similarity analysis

To examine spatiotemporal neural dynamics during touch observation, we correlated time-resolved EEG neural RDMs to fMRI activity RDMs. fMRI data were collected in our previous studies where 37 neurotypical adults viewed the original version of the touch stimuli (3-s video clips) and received positive and negative affective touch during a somatosensory localizer scan (Lee Masson et al., 2018, 2019). Full details on fMRI data acquisition and preprocessing procedure can be found in our previous study (Lee Masson et al., 2018). fMRI data analysis related to the current RSA methods is summarized below.

Neural RDMs of three regions of interest (ROIs), EVC, TPJ/pSTS, and somatosensory cortex, were included in the current study. We chose to focus on these three ROIs to test whether the social-affective meaning of observed touch is extracted first through social perceptual regions (TPJ/pSTS) or through simulation from the somatosensory cortex. Both regions were shown to contain significant information about social touch and be functionally connected in our previous fMRI study (Lee Masson et al., 2018, 2019, 2020). EVC was included as a reference region for early visual processing. For EVC and TPJ/pSTS, visually responsive voxels (i.e., voxels showing increased responses to videos vs rest) were selected within a corresponding anatomic template, i.e., Brodmann area 17 from the SPM Anatomy toolbox (Eickhoff et al., 2005), and TPJ from connectivity-based parcellation atlas (Mars et al., 2012). We name the latter region TPJ/pSTS as the TPJ template includes voxels that are also part of pSTS, and our voxel definition is likely to extract perceptual voxel responses. To define an ROI involved in somatosensory simulation, a separate touch localizer was used to identify voxels that respond to self-experienced affective touch within an anatomic template, Brodmann area 2 from the SPM Anatomy toolbox. Pleasant and unpleasant touch were delivered on the ventral forearm (instead of a hand or a thigh) during this localizer to roughly match the body area involved between receiving and observing touch conditions, as most of the videos show a toucher using their hand to touch a receiver's arm. Identified voxels in the somatosensory cortex represent received touch and thus would represent observed touch through simulation of this process (Lee Masson et al., 2018, 2019). The pairwise correlation between all voxels in each ROI was used to create fMRI neural RDMs and capture the differences in multivoxel neural response patterns between each video pair. A rank correlational method was used to link each participant's time-resolved EEG neural RDMs to each ROI.

**Figure 3.**
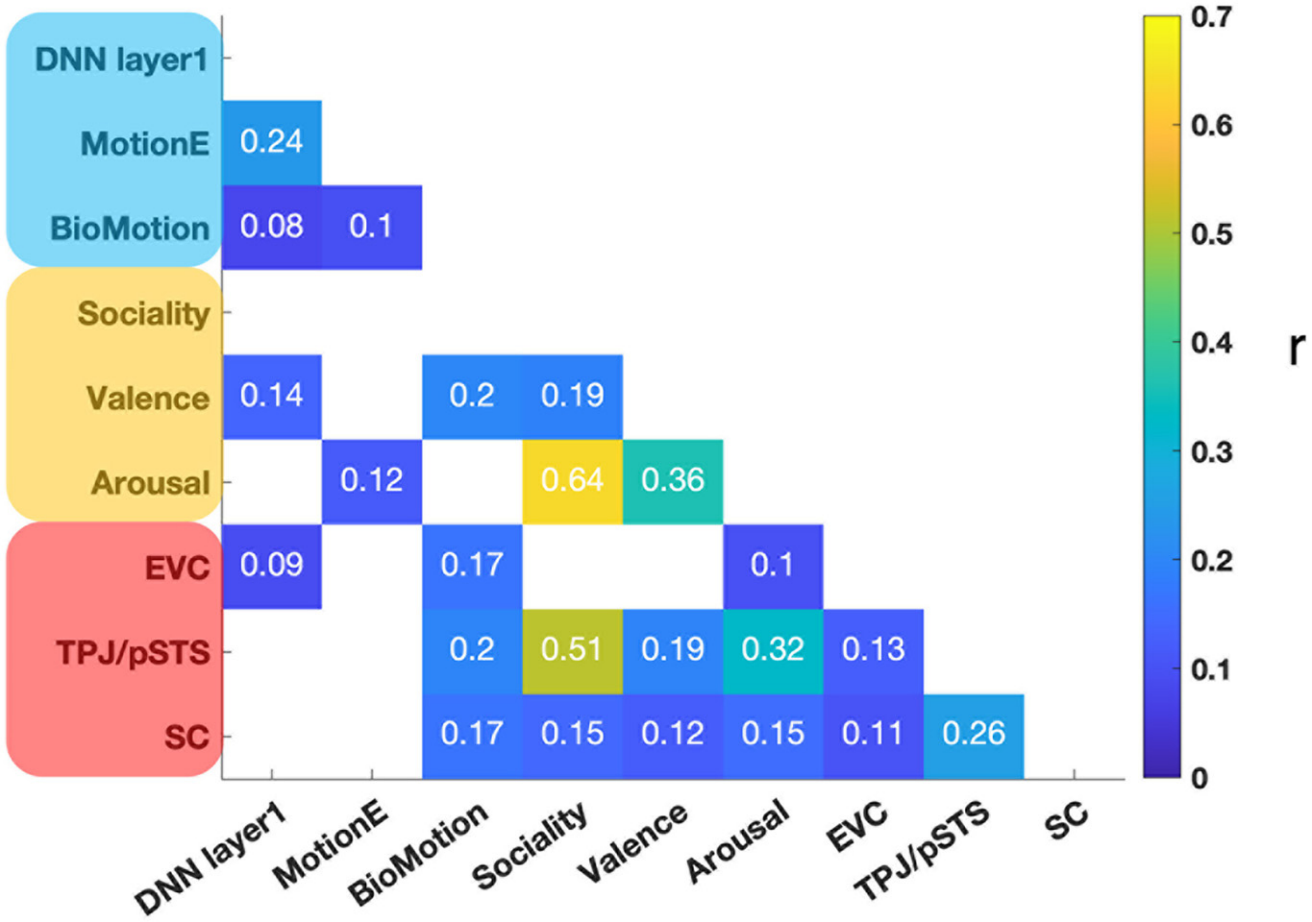
Pairwise correlations between predictors (model RDMs). Three visual (labels colored in blue) and three social-affective (labels colored in orange) features, and fMRI responses of three ROIs (labels colored in red) are included in the RSA. Lower diagonal cells in the matrix contain information about correlation coefficients (*r*) from pairwise comparisons. White colored lower diagonal cells indicate no significant correlation between predictors. No significant negative correlation was observed. DNN = deep neural network, MotionE = motion energy, BioMotion = biological motion, EVC = early visual cortex, TPJ/pSTS = temporoparietal junction/posterior superior temporal sulcus, SC = somatosensory cortex.

Figure 3 shows pairwise correlations between the RDMs of six features and three ROIs from the fMRI data. A strong correlation between sociality and perceived arousal of touch was observed (*r* = 0.64). As found in the previous study (Lee Masson et al., 2018, 2019), TPJ/pSTS strongly represents sociality of observed touch (*r* = 0.51), with this region showing significant neural pattern similarity with the somatosensory cortex (*r* = 0.26).

### Variance partitioning

A time-resolved variance partitioning approach was adopted to examine (1) the unique contribution of the three key brain regions to the EEG signal to characterize the direction of information flow between them, and (2) their shared contribution with the sociality feature to EEG signals. We focus on the sociality feature as TPJ/pSTS and somatosensory cortex both represent the social content of observed touch (Lee Masson et al., 2018, 2019).

To this end, we fit seven different multiple regression models with every possible combination of the three ROIs: EVC, TPJ/pSTS, and somatosensory cortex (i.e., each ROI alone, all three combinations of pairs, and the combination of all three), as well as seven combinations of sociality with the two key ROIs: sociality, TPJ/pSTS, and somatosensory cortex. The time-resolved EEG neural RDM averaged across subjects was the dependent variable in all models. All variables were normalized before regression analysis. Resulting R^2^ values from seven regression models were used to calculate both unique and shared variance. The amount of unique variance explained by each predictor was calculated as follows:

(1) $${\text{UV}}_{\text{POI}}={\text{R}}^{\text{2}}{}_{\text{X}}–{\text{R}}^{\text{2}}{}_{\text{X}-\text{POI}}.$$

R^2^ is a goodness-of-fit measure for the regression model, representing the amount of variance explained by a model consisting of chosen predictors. X reflects three selected predictors included in the model (e.g., brain regions). POI reflects a predictor of interest (e.g., EVC). X-POI reflects the remaining predictors without the predictor of interest (e.g., TPJ/pSTS, and somatosensory cortex). UV _POI_ is the amount of unique variance explained by POI.

The amount of shared variance (SV) explained by every possible combination of selected predictors was calculated as follows:

1. ${\text{SV}}_{\text{123}}={\text{R}}^{\text{2}}{}_{\text{12}}–{\text{R}}^{\text{2}}{}_{\text{2}}+{\text{R}}^{\text{2}}{}_{\text{3}}–{\text{R}}^{\text{2}}{}_{\text{12}}–{\text{R}}^{\text{2}}{}_{\text{13}}–{\text{R}}^{\text{2}}{}_{\text{23}}+{\text{R}}^{\text{2}}{}_{\text{123}}$
2. ${\text{SV}}_{\text{12}}={\text{R}}^{\text{2}}{}_{\text{13}}–{\text{R}}^{\text{2}}{}_{\text{3}}+{\text{R}}^{\text{2}}{}_{\text{23}}–{\text{R}}^{\text{2}}{}_{\text{123}}$
3. ${\text{SV}}_{\text{13}}={\text{ R}}^{\text{2}}{}_{\text{12}}–{\text{R}}^{\text{2}}{}_{\text{2}}+{\text{R}}^{\text{2}}{}_{\text{23}}–{\text{R}}^{\text{2}}{}_{\text{123}}$
4. ${\text{SV}}_{\text{23}}={\text{R}}^{\text{2}}{}_{\text{12}}–{\text{R}}^{\text{2}}{}_{\text{1}}+{\text{R}}^{\text{2}}{}_{\text{13}}–{\text{R}}^{\text{2}}{}_{\text{123}}$

Numbers next to SV and R^2^ denote selected predictors. For example, SV_123_ is the amount of shared variance explained by all three selected predictors. R^2^_12_ is the amount of variance explained by a model consisting of the first and the second predictor.

### Experimental design and statistical analyses

The sample size was determined based on our previous fMRI study that employed the same stimulus set (Lee Masson et al., 2018). EEG neural activity is the within-subject factor and the dependent variable. To explain the EEG data, we included six features and three fMRI ROIs as independent variables. Two additional independent variables (features describing body parts involved in giving and receiving touch) were labeled and included in a *post hoc* analysis. All time-resolved RSA results were tested against chance using a one-tailed sign permutation test (5000 iterations). Multiple comparisons across time were controlled by applying cluster correction with the maximum cluster sum across time windows and an α level of 0.05.

### Data and code accessibility

The current study used analysis code for EEG preprocessing and multivariate analysis used in a previous study (Dima et al., 2022), available on GitHub (https://github.com/dianadima/mot_action/tree/master/analysis/eeg). The code for stimulus presentation and ERP analysis is available on GitHub (https://github.com/haemyleemasson/EEG_experiment). EEG data are available at https://osf.io/5ntcj/.

## Results

### Early evoked response differences between social and nonsocial touch

**Figure 4.**
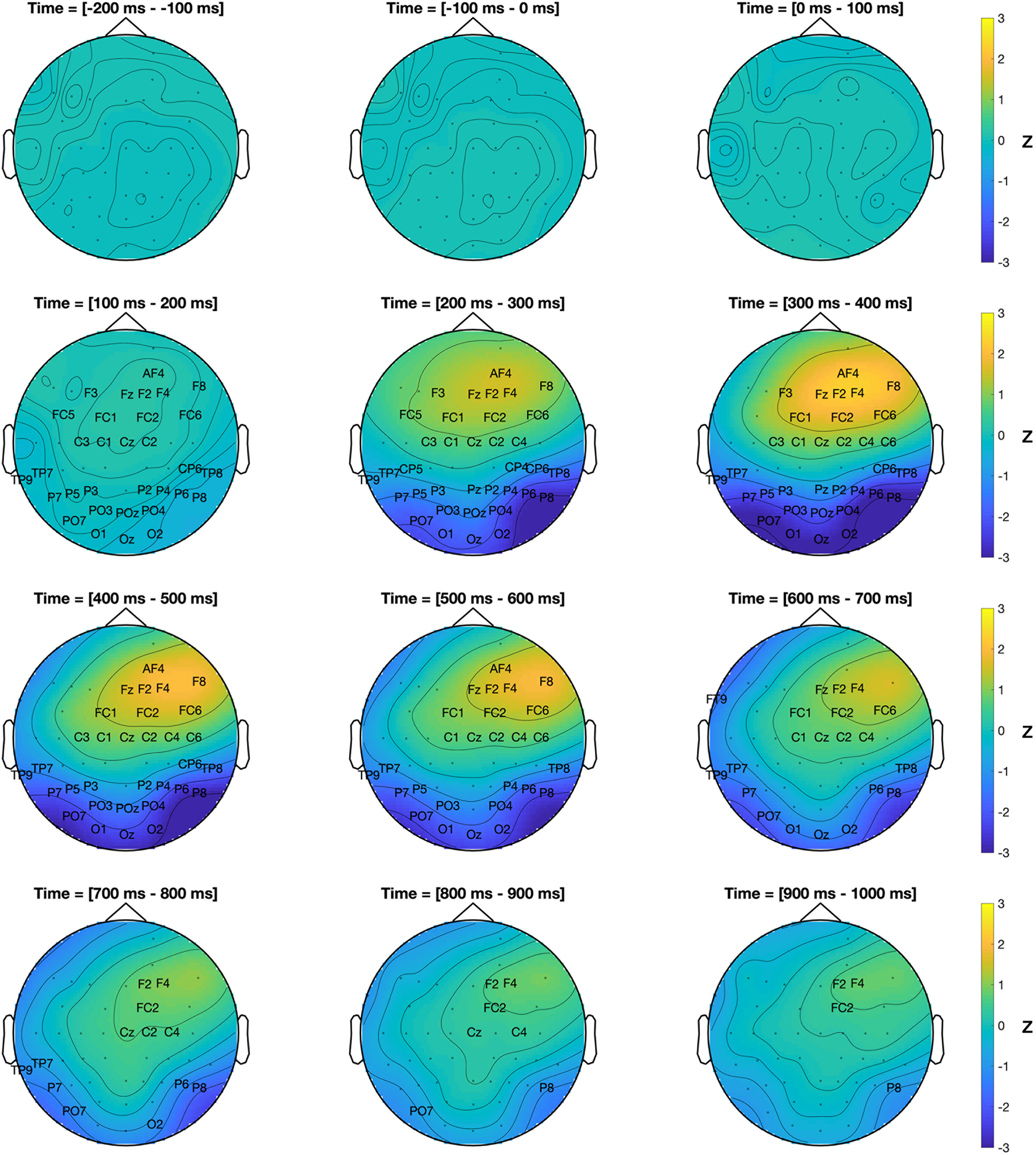
ERP differences between social and nonsocial touch observation. Topographical scalp maps show the social > nonsocial contrast, averaged across successive 100-ms time slices. Positive *z* scores (increased ERP for social touch) are shown in yellow and negative *z* scores (increased ERP for nonsocial touch) are shown in blue. Channels showing significant differences in ERPs were labeled with channel names in the scalp map. Channels that did not reach statistical significance were marked with dots to indicate their locations on the scalp.

We compared the ERPs evoked by observed social and nonsocial touch events to measure the effect of sociality on the magnitude of ERPs for each channel and time window. We observed widespread differences across the scalp, beginning at 100 ms postvideo onset. Anterior sensors showed enhanced activation during social touch observation (Fig. 4, yellow regions), whereas activations were stronger at the posterior sensors during nonsocial touch observation (Fig. 4, blue), perhaps because of the presence of objects in these videos. In particular, in most of the time windows, observing social touch evoked the strongest activation at channel F2, located approximately near superior frontal gyrus (Scrivener and Reader, 2022), whereas P8, located near lateral occipital cortex, was most activated in nonsocial touch. These ERP results indicate that the sociality of video clips affects activations even at early stages of processing.

### Social-affective features are processed shortly after visual features

**Figure 5.**
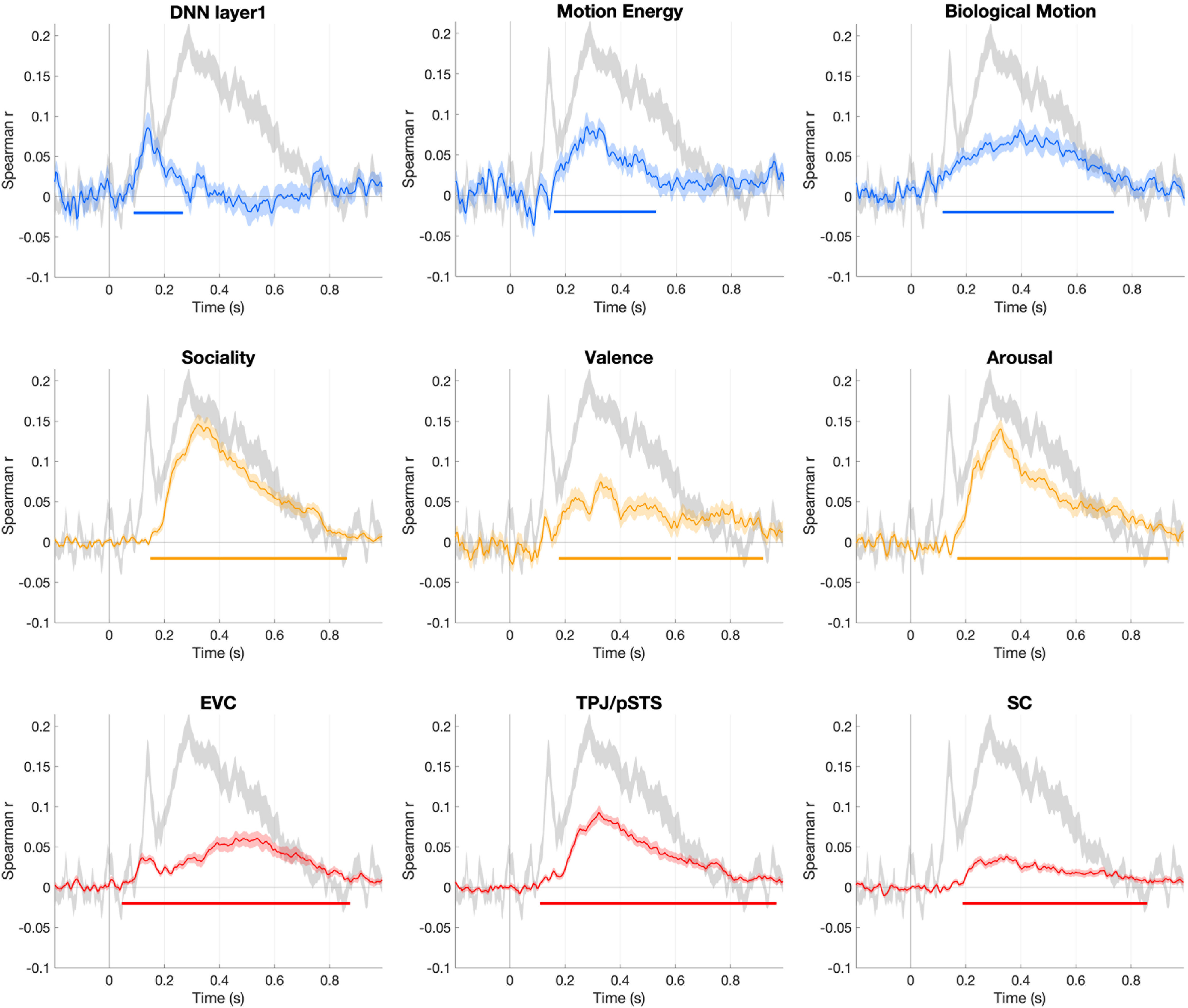
The time courses of all predictor correlations with EEG data (mean ± SEM shown in light colors). This figure shows group-level spearman correlations between visual (time courses shown in blue, top panel), social-affective (time courses in yellow, middle panel), and fMRI activity predictors (time courses in red, bottom panel) and the time-resolved EEG neural patterns. The colored horizontal line in each plot marks significant time windows where observed correlation coefficients are significantly greater than 0 (one-tail sign permutation for the group-level statistics, cluster-corrected *p* < 0.05). The noise ceiling, quantified as leave-one-subject-out correlation, is shown in light gray (mean ± SEM).

Using time-resolved RSA, we evaluated the neural dynamics of touch observation or how quickly different stimulus features are processed in the brain. To this end, we correlated three visual and three social-affective features to each participant's EEG neural patterns. A group-level RSA revealed that low-level visual features captured by the first layer of AlexNet correlated significantly with EEG neural patterns beginning at 90 ms poststimulus onset, followed by biological motion and motion energy at 120 and 160 ms, respectively (Fig. 5, top). Strikingly, social-affective features correlated with EEG neural patterns shortly after visual features, beginning at 150 ms postvideo onset for sociality, 170 ms for arousal, and 180 ms for valence (Fig. 5, middle). All six features were spontaneously extracted in the brain as participants were not directed to any of these features during the EEG experiment.

**Figure 6.**
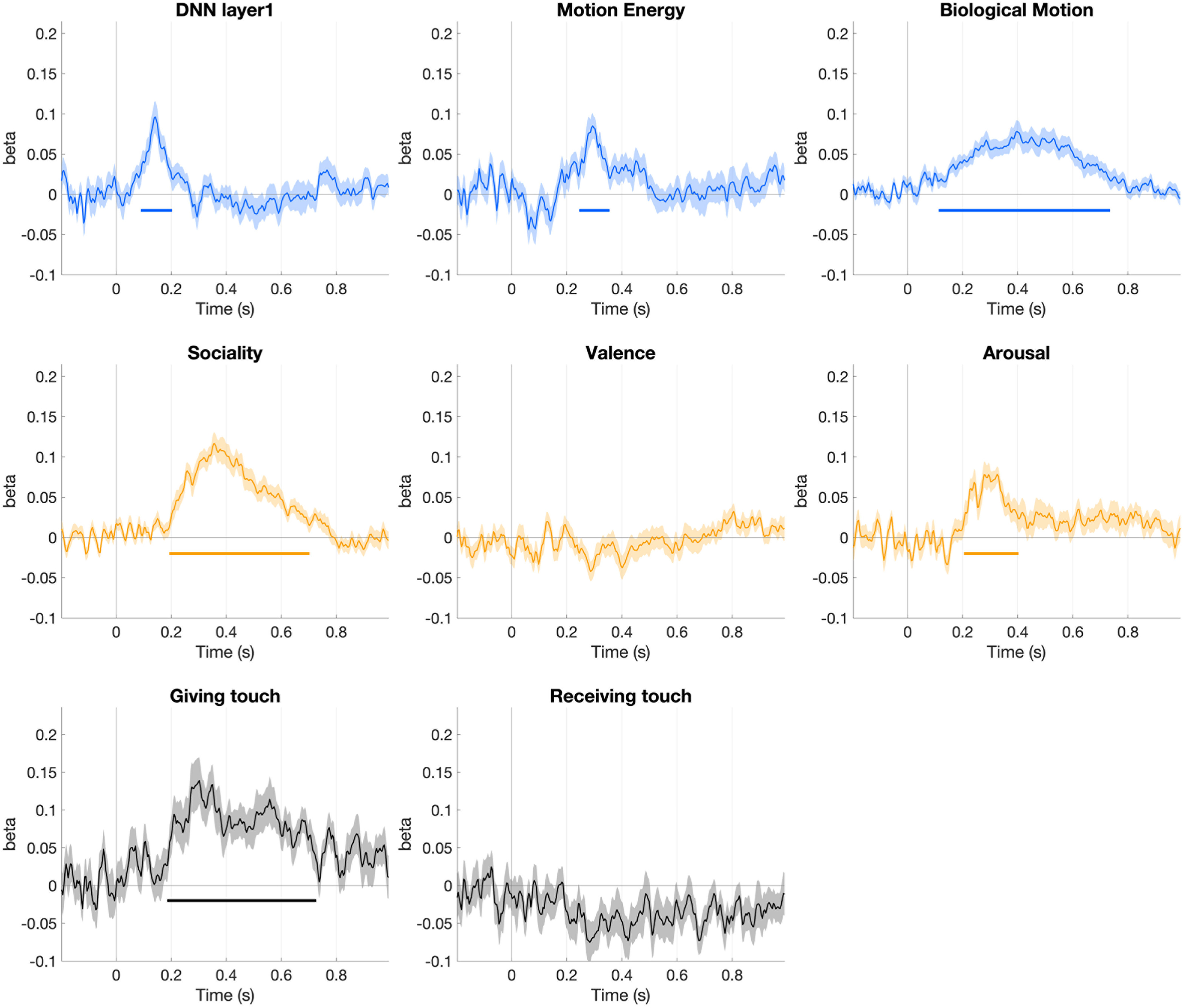
The time courses of each feature predicting EEG neural patterns in a multiple regression model (mean ± SEM shown in light colors). This figure shows group-level regression β coefficients describing the relationship between visual (time courses shown in blue, top panel), social-affective (time courses in yellow, middle panel), and the time-resolved EEG neural patterns. The time courses of two features (the body parts involved in giving and receiving touch) predicting EEG data in a separate multiple regression model are shown in the bottom panel (time courses in black).

In a follow-up analysis, we fit a multiple regression model to determine the extent to which each feature explained the time-series EEG neural patterns while accounting for variance explained by other features. The results of the multiple regression analysis aligned with those obtained from the rank correlational analysis. Specifically, low-level visual features, biological motion, and motion energy started to explain the time-series EEG neural patterns at 90, 120, and 250 ms following the onset of the video, respectively (Fig. 6). Sociality and arousal of the video explained time-series neural patterns immediately after the visual features, beginning at 200 and 210 ms, respectively. In contrast to the findings from rank correlation analysis, the valence feature did not explain the EEG data once the effects of other features were controlled for. Nonetheless, our overall conclusion remains unchanged: social-affective features, namely, sociality and arousal, are processed shortly after visual features.

Lastly, in a *post hoc* analysis, we fit a multiple regression model to determine whether two features describing the body parts involved in giving and receiving touch explained the time-series EEG neural patterns during the social touch condition (Fig. 6). The feature capturing which body part was involved in giving touch started to explain the time-series EEG neural patterns at 190 ms following the onset of the video. Surprisingly, the feature capturing which body part was involved in receiving touch did not explain the time-series EEG neural patterns.

### EEG neural patterns correlate with responses first from early visual cortex, then TPJ/pSTS, and finally somatosensory cortex

We tracked the spatial-temporal neural dynamics of touch observation using EEG-fMRI fusion methods to examine the information flow between EVC, TPJ/pSTS, and somatosensory cortex. These results show a clear order between the neural latencies of each ROI. We find that neural information first arises in early visual cortex 50 ms postvideo onset, and then in TPJ/pSTS at 110 ms, and finally in somatosensory cortex at 190 ms (Fig. 5, bottom). A Mann–Whitney *U* test showed significant differences in the onset latencies of all three combinations of pairs (*p* < 0.001).

### Visual and social perceptual, but not somatosensory, brain regions explain unique variance in EEG signals during touch observation

**Figure 7.**
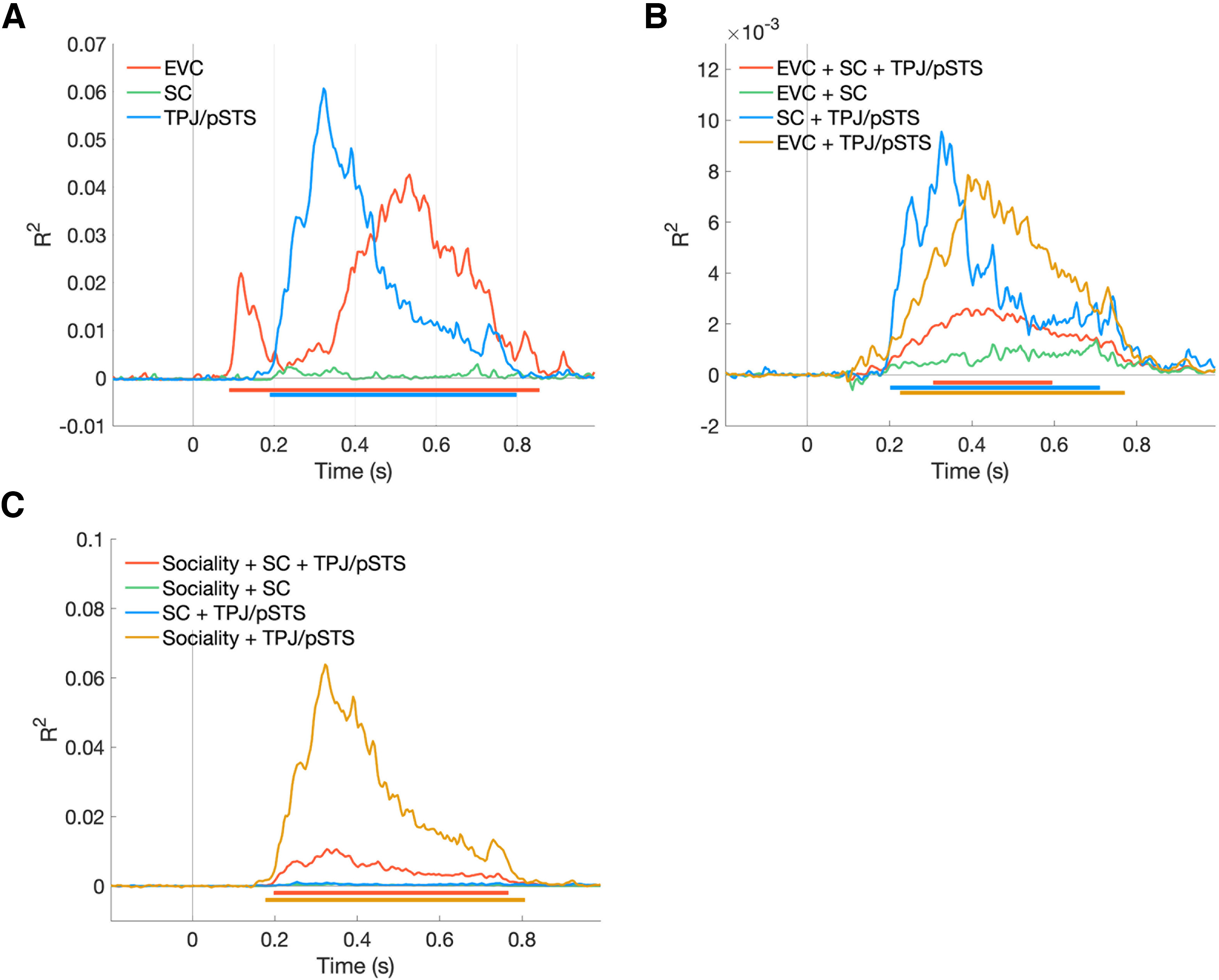
Time-resolved variance partitioning results. Multiple regressions were performed with the time-resolved EEG RDM averaged across subjects as a dependent variable. ***A***, The unique contributions of the fMRI activity in three brain regions with EEG data. ***B***, The shared contributions of the three brain regions with EEG data. ***C***, The shared contributions of TPJ/pSTS and somatosensory cortex (SC) with the sociality feature. The horizontal line in each plot marks significant time windows (one-tailed sign permutation for the group-level statistics, cluster-corrected *p* < 0.05).

Given the significant correlation between neural patterns in TPJ/pSTS and those in somatosensory cortex (Fig. 3), it is unclear whether the time-resolved correlations between those brain responses and EEG signals (Fig. 5, bottom panel) are driven by shared or unique variance across different brain regions. Thus, we examined unique and shared contribution of fMRI responses in each region to EEG neural patterns using variance partitioning analysis to further characterize the information flow across the brain regions involved in touch observation. Variance partitioning revealed that EEG neural patterns are uniquely explained by EVC at 94 ms after video onset, then by TPJ/pSTS at 190 ms (Fig. 7*A*). Importantly, somatosensory cortex activity did not explain any unique variance in the EEG data, but shared variance with TPJ/pSTS beginning at 206 ms (Fig. 7*A*,*B*). There was also shared variance between EVC and TPJ/pSTS at ∼100 ms after the initial onset of EVC, suggesting feedback from the social brain to EVC at later time points (Fig. 7*B*).

### Temporally resolved information in TPJ/pSTS explains unique variance in social touch observation

Lastly, we asked whether and when the sociality of observed touch, the feature that explains EEG data the most strongly, shares variance with TPJ/pSTS or with somatosensory cortex activity. We find that TPJ/pSTS responses share variance with sociality in explaining the EEG data at 180 ms after video onset (Fig. 7*C*). In contrast, activity in somatosensory cortex alone does not share variance with sociality. TPJ/pSTS, somatosensory cortex, and sociality do explain shared variance with EEG signals, but this is substantially later and weaker than variance explained by TPJ/pSTS and sociality alone. Together, these results suggest little direct involvement of somatosensory cortex in representing the sociality of observed touch.

## Discussion

We examined the spatiotemporal neural dynamics of social touch observation. Combining time-resolved RSA, fMRI-EEG fusion, and variance partitioning analyses (Fig. 2), we for the first time identified the speed at which each feature of observed touch is processed (Figs. 5, 6), as well as the direction of information flow between brain regions. Our results revealed a pathway between early visual processing and social perception, with only later involvement of somatosensory simulation (Fig. 7).

### Early processing of social-affective meaning of observed touch

Observing physical contact between two individuals resulted in greater neural activity in the frontal regions, while observing an individual touching an object led to stronger neural activity in the occipital regions (Fig. 4). These findings are consistent with previous neuroimaging studies showing differential neural responses to social and nonsocial touch (Peled-Avron et al., 2016; Peled-Avron and Shamay-Tsoory, 2017; Lee Masson et al., 2018, 2019). Similar to previous work, the current study suggests that observing two individuals exchanging touch involves social-cognitive mechanisms, such as mentalizing and emotion recognition (Peled-Avron and Shamay-Tsoory, 2017; Lee Masson et al., 2018, 2020; Schirmer and McGlone, 2019; Arioli and Canessa, 2019), whereas recognizing a touched object requires additional visual processing (Goodale et al., 1994; Lee Masson et al., 2018, 2020; Wurm and Caramazza, 2022). Concerning the processing speed, ERPs evoked by social touch were distinguishable from those elicited by nonsocial touch as early as 100 ms after stimulus onset. Overall, the current study extends earlier findings by revealing that the neural distinction between social and nonsocial touch is established at an early stage in neural processing.

A classical ERP method is analogous to a univariate approach in fMRI, in that both methods unveil the extent to which a channel or voxel is activated in response to a given stimulus. However, this univariate method does not provide a comprehensive account of how the configuration of multiple channels evolves over time or how stimulus features are represented in the spatial patterns (Davis et al., 2014; Pillet et al., 2020). To answer this question, we employed time-resolved RSA to link EEG spatial patterns to visual and social-affective features characterizing a touch event (Fig. 2). Time-resolved RSA revealed that social-affective information, whether touch is social, pleasant, or emotionally intense, is processed rapidly and immediately following the processing of low-level visual features, motion, and body movements (Fig. 5).

To date, no studies have used time-resolved RSA to investigate the temporal dynamics of neural responses during touch observation. Nonetheless, our findings on the speed of visual processing during touch observation are consistent with those reported in other areas of vision research. We found that the low-level visual features extracted with the first layer of AlexNet explain EEG neural patterns early, beginning at 90 ms after stimulus onset. This finding aligns with previous work investigating scene perception and action observation using similar methods (Cichy et al., 2017; Dima et al., 2022). The perception of biological motion emerges early as well, beginning at 120 ms. The onset latency observed in the current study is consistent with the timing of neural processing reported in other studies of biological motion and action perception (Oram and Perrett, 1994; D.H.F. Chang et al., 2021; Dima et al., 2022). It is important to clarify that the term “biological motion” here does not refer to action categories. Rather, it refers to the perception of body movements. As an example, body movements required for different actions, such as hugging a person or carrying a box, are perceived similarly. Regarding motion energy, since we rely on basic optic flow techniques to calculate motion and the present study is not designed to focus on motion energy, we remain cautious in providing detailed interpretation of our results (refer to Bergen and Adelson, 1985; Dima et al., 2022). While we have addressed the processing speed of three visual features, there are other high-level visual features to consider, such as which body part was used to initiate touch or which body part was touched. Our *post hoc* analysis revealed that representations of the body part used to give touch emerge shortly after visual processing, beginning at 190 ms. Surprisingly, the body part used to receive touch did not explain significant variance in the EEG signal. This finding potentially implies that viewers might direct their attention toward the touch initiator, thereby interpreting the meaning of social touch. However, the current stimulus set mostly focuses on the arms and hands and not designed to answer this question. Creating a novel social touch stimulus set that includes a wider variety of body parts may help better address this.

We found that the onset latency for processing sociality, arousal, and valence information of observed touch occurred within a time frame of 150–180 ms. This finding aligns with previous research that has demonstrated the early processing of perceived valence (145 ms) and arousal (175 ms) in a variety of emotional images (Grootswagers et al., 2020). Note that valence no longer explained EEG neural patterns once the effects of other features were accounted for. This may be because the valence feature correlated with other features, such as arousal and biological motion. Furthermore, the strength of the rank correlation between valence and time-series EEG neural patterns was relatively moderate in contrast to other features. These two factors likely contributed to the absence of significant findings when employing a multiple regression model.

The processing speed of social-affective information varies and may be influenced by the level of complexity and naturalness of the stimuli used. Sociality information from simple, well-controlled images is processed rapidly, as indicated by changes in P100 components (Peled-Avron and Shamay-Tsoory, 2017), whereas natural stimuli tend to require more time to process (Isik et al., 2020; Dima et al., 2022). The stimulus set used in this study focused on body movement without other contextual information about touch, which may explain the fast processing times. Further, the sociality model in the current study distinguishes between touch directed toward a person and touch directed toward an object, and as a result, also captures the presence of either two people or one person in the scene. Further research is needed to investigate the speed at which social-affective information is extracted in more ecologically valid and complex settings, and to explore how the neural dynamics of social touch interaction differ from social interaction without touch or interaction where two people touch an object together. Together, the ERP and RSA results presented here show that the brain detects the social-affective significance of touch at an early stage, well within the timeframe of feedforward visual processing (Lamme and Roelfsema, 2000). These results highlight the importance of touch perception as a fundamental aspect of the human visual experience.

### The social-affective meaning of touch is initially extracted through a social perceptual pathway

With time-resolved RSA and variance partitioning analyses, we determined the direction of information flow between EVC, TPJ/pSTS, and somatosensory cortex. We demonstrated that the social-affective meaning of touch is initially extracted through a social perceptual pathway, followed by the subsequent involvement of somatosensory simulation. These findings suggest that touch observation is not mediated by the reenactment of somatosensory representations, which contradicts the embodiment theory of social perception (Rizzolatti and Sinigaglia, 2010, 2016). Instead, the somatosensory cortex appears to receive information from a social perceptual pathway, as indicated by this region's absence of unique variance with EEG neural activity.

Although somatosensory involvement occurs at a later stage, its contribution to social understanding should not be undervalued. Individuals who struggle with social interaction, particularly in the context of interpersonal touch, tend to exhibit weak or absent somatosensory activity, which can decrease social bonding (Peled-Avron and Shamay-Tsoory, 2017; Lee Masson et al., 2018, 2019). The relay of social information from TPJ/pSTS to the somatosensory cortex may help individuals empathize with others and comprehend another person's emotional states at a more profound level (Schaefer et al., 2012, 2013; Bolognini et al., 2013; Peled-Avron et al., 2016; Peled-Avron and Woolley, 2022). The methodology presented here provides an opportunity for future work to examine whether the lack of somatosensory simulation in particular groups of individuals is associated with inadequate information flow between social perceptual and somatosensory system.

In conclusion, our study, for the first time, revealed that social-affective information of observed touch is processed rapidly and directly through social perceptual brain regions. Positive touch plays an important role in establishing and maintaining social bonds (Hertenstein et al., 2006b; Chatel-Goldman et al., 2014; Suvilehto et al., 2015). It facilitates effective communication, fosters greater trust and empathy, and provides relief from stress, anxiety, and pain, ultimately leading to improved psychological and physical wellbeing (Debrot et al., 2013; Korisky et al., 2020; Shamay-Tsoory and Eisenberger, 2021; Packheiser et al., 2023). While social touch is often associated with positive social interactions, touch can be used as a potent tool for aggression (Hertenstein et al., 2006a; Lee Masson and Op de Beeck, 2018). Negative touch, such as physical assault, can result in physical pain or emotional distress for a receiver, potentially leading to adverse health outcomes (De Puy et al., 2015; Stubbs and Szoeke, 2022). Thus, recognizing the dual nature of touch seems essential for humans. Our findings suggest that the early neural processing of social and emotional signals conveyed through touch may play a key role in the successful use of social touch during rapidly-changing, real world social interactions.
